# Factors associated with perceived caregivers' willingness to provide care among older adults with disabilities in China

**DOI:** 10.3389/fpubh.2023.1170594

**Published:** 2023-06-05

**Authors:** Chang Sun, Dijuan Meng

**Affiliations:** School of Nursing, Nanjing University of Chinese Medicine, Nanjing, China

**Keywords:** informal caregiving, willingness to care, disability, older adults, long-term care

## Abstract

**Objective:**

The aim of this study was to analyze the status quo and associated factors of care recipients' perceptions of caregivers' willingness to provide care among disabled older adults in China. Thus, this study contributes to our understanding of vulnerable older populations who are at a high risk of receiving support from informal caregivers who are unable or unwilling to take the caregiver role.

**Methods:**

We analyzed the cross-sectional data of 3,539 disabled older adults who received informal care at home from the seventh wave of the 2018 Chinese Longitudinal Healthy Longevity Survey (CLHLS). Multiple logistic regression models were used to examine the variables associated with the respondents' perceived caregivers' willingness to care from five aspects: respondents' sociodemographic attributes, health-related data, family endowment, access to health care services and community-based long-term care services (CBLTCS).

**Results:**

This study found that the majority of disabled older adults (90.9%) had a positive attitude toward their caregivers' willingness to care and the care they received; however, 7.0% of the adults were concerned about their caregivers' ability to handle the care. Moreover, there was a small number of disabled older people (2.1%) who felt that their caregivers were reluctant to care or lacked patience. The results from the multiple logistic regression showed that disabled older adults with socioeconomic disadvantages (living in rural areas, being poor and with no children who frequently visited) or high demand (with severe disabilities or cognitive impairment) were more likely to consider that their caregivers needed respite care. Those adults with anxiety symptoms, a lower amount of care time, poor self-rated financial status and poor accessibility to health care services were more likely to report that their caregivers were reluctant to administer care.

**Conclusion:**

This study found that living in rural areas, being poor, with no children who frequently visited, severe disabilities or CI were positively associated the care recipients' perception that caregivers needed respite care. While anxiety symptoms, a lower amount of care time, poor self-rated financial status and poor accessibility to health care services were significantly associated with care recipients' perception of caregivers' reluctance to care. Our findings highlight the awareness of monitoring informal carers' willingness to care or capability to enact caring tasks.

## 1. Introduction

Due to declining fertility rates and increasing life expectancy, China is experiencing accelerated population aging. By the end of 2022, the population of China aged 65 years or over was 209.78 million, accounting for 14.9% of the national population (1), which indicates that China has become an aged society and is now gradually progressing into a superaged society (2). In addition to the rapidly aging population, the number of older adults with disabilities is also increasing. It is predicted that the number of disabled older people is projected to increase from 52.71 million in 2020 to ~78 million in 2030 in China (3). Moreover, the years lived with disability (YLDs) of older adults in China were estimated to increase from 5.78 in 2015 to 7.44 years in 2030 (3). In such cases, the manner in which adequate long-term care (LTC) services can be provided for the ever increasing number of older people with care dependency has become a prominent issue in China.

To address this concern, LTC insurance (LTCI) was introduced in China in 2016, with 15 cities selected as the first batch of LTCI pilots (4). Currently, this insurance covers 49 cities and 145 million people across the country, with a focus on providing formal support to older adults with moderate and severe disabilities to ensure their basic daily life assistance (5). However, for the large number of disabled people, the coverage of policy-based LTCI is still insufficient. Moreover, due to being in the initial stage of LTCI policy implementation, it still faces challenges (such as the inefficient delivery of LTC services), which results in the limited substitution of publicly provided formal care for informal care. Informal care was defined as the provision of unpaid care to a family member, relative, friend or neighbor with disabilities in the study.

The majority of older adults wish to “age in place” for as long as possible, even when they face functional limitations (6). This scenario is especially true in China, which has long been influenced by Confucianism and possesses filial piety as a core virtue of society. With the traditional concept of “Raise children for old age,” people in China prefer to be cared for by their adult children at home in their older years. Therefore, family-based informal care remains an important pillar of the LTC system in China and will continue to be so in the future. It has been reported that ~90% of disabled older adults are cared for exclusively by family members without any support from social services outside of the home (7). However, with the shrinking household size and the influence of westernization and urbanization, the informal care system in China has become vulnerable.

Despite decades of innovative research in the domain of informal care, the majority of studies to date have focused on a limited view of the care-giving experience, with a primary focus on the aspects of care burden, stress (8–11) and measures or coping strategies to reduce these factors (12–14) to maintain carers in their roles. However, caregiving willingness, which has been defined as a caregiver's attitude toward providing emotional, instrumental and nursing support for an individual (15), was not a common issue in previous research. Limited studies have examined the hypothetical willingness of individuals to provide informal care if such a need arises in the future (16, 17), as well as willingness to provide care for specific patient groups [e.g., AIDS patients (15) and multiple sclerosis patients (18), among other patients] and the caregiving willingness of subsample carers [e.g., male carers (19), spouse carers (18) and low-income female carers (20), among other carers].

Apart from the burden of caring, caregiving willingness is a response to actual or perceived burden, which may be exhibited by caregivers with willingness or reluctance (21). Due to the fact that the entire LTC system cannot function without the commitment of informal caregivers, it seems to be meaningless to put this resource at risk by identifying those individuals who are not willing to assume the role. Given that caring for a disabled family member is a time-intensive and labor-intensive activity with increasing demands (22), reluctance to care would be understandable. Therefore, it would be unwise to ignore the reality that people may feel differently about being a family caregiver, with attitudes ranging from being highly committed to being unwilling to care. Otherwise, the consequences of ignoring the reality cannot be ignored, as it may generate adverse outcomes for care recipients, caregivers and the LTC system. Prior research has identified undesirable results of caregiving reluctance, including deterioration in caregiver-care recipient relationships (23, 24), reduced quality of care (23, 25) and institutionalization (26).

Furthermore, in previous studies, willingness to care was solely described from the perspective of caregivers. However, the actual view of caregivers may be hidden to avoid censure because of the pressure of social norms (21), especially in the context of China's filial piety culture. Moreover, older adults with disabilities play a very significant role in shaping caregivers' willingness to care (18). Thus, this study aimed to analyze the status quo and factors associated with perceived caregivers' willingness to provide care in China from the perspective of disabled older adults who received informal care at home, thus contributing to our understanding of vulnerable populations who are at a high risk of receiving support from family caregivers who are unable or unwilling to take the role.

## 2. Materials and methods

### 2.1. Data sources

The Chinese Longitudinal Healthy Longevity Survey (CLHLS) is a nationwide prospective cohort study. The first wave was established in 1998, and subsequent follow-up surveys were conducted in 2000, 2002, 2005, 2008, 2011, 2014, and 2018. The survey respondents were randomly selected through a multistage cluster sampling approach from 23 out of 31 provinces (22 out of 31 provinces before 2008) in China. The CLHLS aims to investigate factors associated with healthy longevity in humans, which covers many types of information, including respondents' sociodemographic characteristics, health status, social activity and lifestyles. Data were collected by trained interviewers using a structured questionnaire in participants' homes. The study was authorized by the Ethics Committee of Peking University (IRB00001052-13074), and written informed consent was obtained from all of the participants and/or their families.

**Figure 1:**
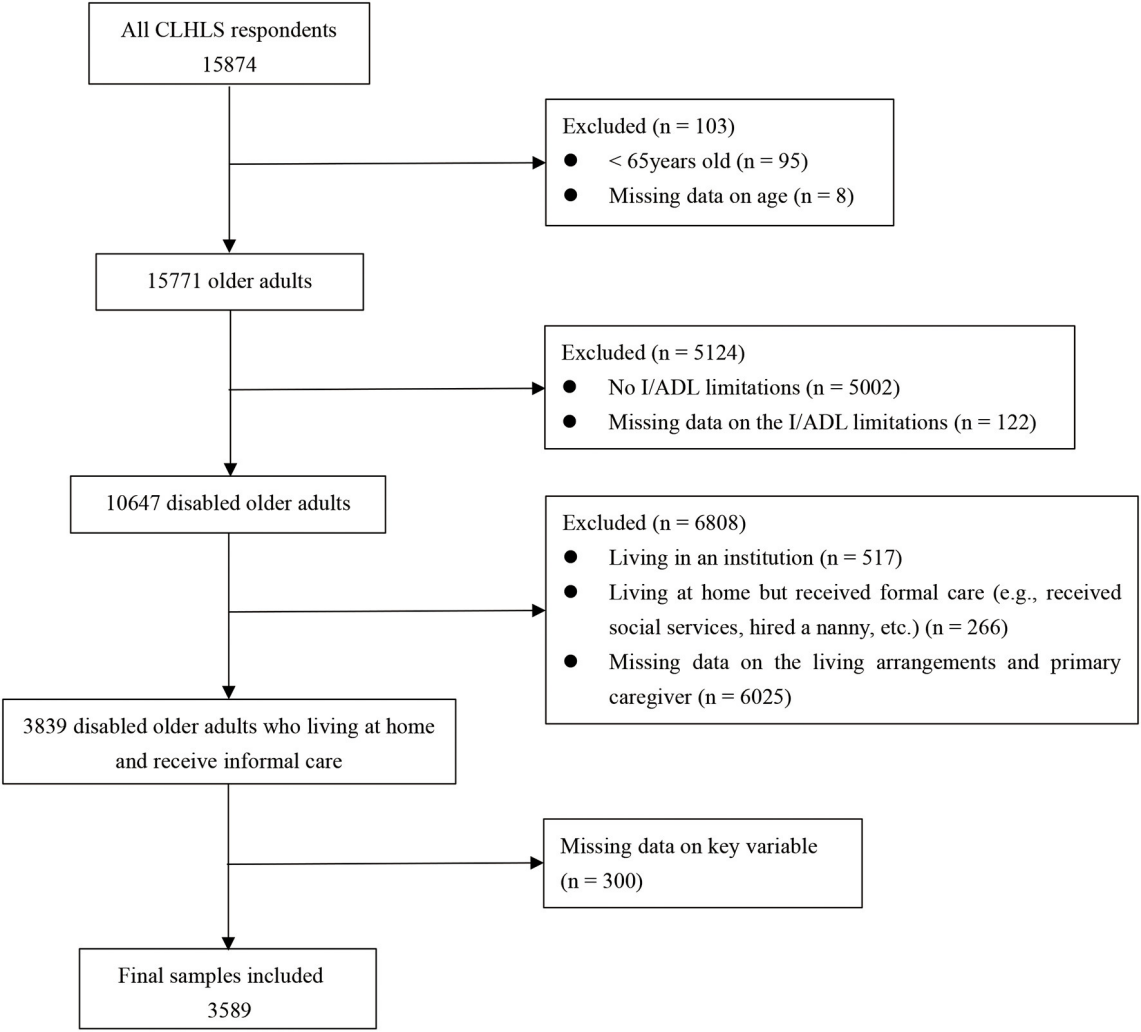
Flow chart of the study sample.

The data used in this study were derived from the seventh wave of the CLHLS in 2018, which contains 15,874 participants. Due to the fact that our goal was to investigate perceived caregivers' willingness to provide care among community-dwelling disabled older adults and their associated factors, three inclusion criteria were imposed on the study sample: respondents (a) should be 65-years-old or above; (b) should have at least one I/ADL limitation; and (c) should live at home and receive informal care provided by their spouses, children, relatives, friends and neighbors. Figure 1 shows how participants in the current study were selected. Finally, 3,539 eligible people were included as the research sample for the present analyses.

### 2.2. Measures

#### 2.2.1. Dependent variable

We considered perceived caregivers' willingness as the dependent variable, which was assessed by using the question “what do you think of the caregiving performance of your primary caregiver?” in the 2018 CLHLS survey. Possible responses were coded as a categorical variable into three groups: 1 = willing to and provide adequate care; 2 = willing to but need respite care; and 3 = unwilling to care or without patience.

#### 2.2.2. Independent variables

According to previous studies and considering currently available data in the 2018 CLHLS survey, a total of 20 factors were conceptualized as the independent variables, including older adults' sociodemographic characteristics, health-related data, family endowment, access to health care services and community-based long-term care services (CBLTCS).

Sociodemographic attributes included age (aged 65–79 years = 1, 80–99 years = 2 and ≥ 100 years = 3), gender (male = 1), place of residence (city/town = 1), education level (illiterate = 1, primary school = 2 and junior high school and above = 3), marital status (currently married = 1) and financial independence (yes = 1).

Health-related data were measured by using five indices, including self-rated health, depression, anxiety, cognitive function and I/ADL limitations. Self-rated health was assessed by asking participants how they rated their general health status, ranging from “very good” to “very poor” (5 points) (very good/good = 1, average = 2 and poor/very poor = 3). Depression was measured by using the Center for Epidemiologic Studies Depression Scale Revised-10 (CESD-R-10), with a score of 10 or above indicating depression (6, 27) (with depression = 1). Anxiety was measured by using the Generalized Anxiety Disorder 7-item scale (GAD-7), and a cutoff of 5 indicated anxiety (28, 29) (with anxiety = 1). Cognitive function was assessed by using the Chinese version of the Mini-Mental State Examination (MMSE), and scores ranged from 0 to 30, with a higher score indicating better cognitive function. We adopted the education-based criteria to define “cognitive impairment (CI)” (with CI = 1): ≤ 17 for those illiterate individuals, ≤ 20 for those individuals with 1 to 6 years of education and ≤ 24 for those individuals with more than 6 years of education (30, 31). Additionally, I/ADL limitations were assessed by asking participants about their difficulty in performing six basic ADLs (bathing, dressing, toileting, indoor transferring, continence, and eating) and eight IADLs (visiting neighbors, going shopping, cooking meals, doing laundry, walking one kilometer, carrying 5 kg weight, crouching and standing 3 times and taking public transportation). We coded I/ADL limitations as a categorical variable into three levels: mild I/ADL limitations (only IADL limitations) = 1, moderate I/ADL limitations (1 or 2 ADL limitations) = 2 and severe I/ADL limitations (3 or more ADL limitations) = 3 (32).

Family endowment factors included self-rated family financial status (very rich/rich/average = 1, poor/very poor = 0), annual household income (logarithm; continuous numerical variable), number of cohabiting family members (continuous numerical variable), number of children who had frequently visited (no children = 1, one child = 2 and more than one child = 3), primary caregiver (spouse = 1, son/daughter-in-law = 2, daughter/son-in-law = 3 and other people = 4), hours of care received in the past week (≤ 24 h = 1, 25–96 h = 2 and 97–168 h = 3) and cost of care in the past week (logarithm; continuous numerical variable).

Access to health care services was assessed by using the question: “Could you get adequate medical services when it is necessary (yes = 1)?” Access to CBLTCS was measured by asking the participants whether eight types of CBLTCS and other services were provided in their community. Subsequently, we calculated the sum of the types of provided services (continuous numerical variable).

### 2.3. Statistical analysis

Descriptive statistics were calculated by using percentages for the categorical variables and means and standard deviations (SDs) for the continuous variables. Chi-square tests, analysis of variance (ANOVA) and Kruskal–Wallis nonparametric tests were used to test the differences in the distribution of respondents' perceived caregivers' willingness to provide care by their characteristics, as appropriate. With “willing to and provide adequate care” as the reference group, multiple logistic regression models were applied to analyze the associated factors related to perceived caregivers' willingness to provide care among older adults with disabilities. All of the analyses were performed by using SPSS 26.0 software (SPSS Inc., Chicago, IL, USA). The results were considered to be statistically significant at a *p* < 0.05.

## 3. Results

Table 1 displays the characteristics of the study respondents. Although the vast majority of the respondents (90.9%) felt that their caregivers were willing to and took good care of them, 7.0% of the respondents thought that their caregivers were willing to care for them but needed respite care, and 2.1% of the respondents perceived that their caregivers were unwilling to provide care or lacked patience. Most of the respondents were oldest-old (aged 80+ years), approximately two-thirds of the respondents were female and more than half of the respondents lived in cities/towns due to China's accelerated urbanization process. In addition, most of the respondents (63.1%) did not receive any formal education, the proportion of respondents who were currently in a marriage was relatively small (18.4%) and the majority of eligible older individuals (80.1%) failed to achieve financial independence. More than half of the respondents rated their general health status as being not poor (61.2%); however, 24.8%, 12.5%, and 55.9% of the respondents reported having depression, anxiety and CI, respectively. Regarding I/ADL limitations, 13.1% of the respondents had mild I/ADL limitations, 40.8% of the respondents had moderate I/ADL limitations and 46.1% of the respondents had severe I/ADL limitations.

**Table 1:** The characteristics of the study respondents.

**Variable**	**Variable value**	***N* (Mean)**	**Percentage (SDs)**
**Dependent variable**			
Perceived caregivers' willingness	willing to and provide adequate care = 1	3,218	90.9%
	willing to but need respite care = 2	248	7.0%
	unwilling to care or without patience = 3	73	2.1%
**Independent variables**			
**Sociodemographic attributes**			
Age	65–79 = 1	256	7.2%
	80–99 = 2	1,781	50.3%
	≥ 100 = 3	1,502	42.4%
Gender	male = 1	1,156	32.7%
	female = 0	2,383	67.3%
Place of residence	city/town = 1	1,978	55.9%
	rural = 0	1,516	44.1%
Education level	illiterate = 1	2,233	63.1%
	primary school = 2	1,055	29.8%
	junior high and above = 3	251	7.1%
Marital status	currently married = 1	643	18.4%
	divorced/widowed/single = 0	2,861	81.6%
Financial independence	yes = 1	687	19.9%
	no = 0	2,768	80.1%
**Health-related data**			
Self-rated health	very good/good = 1	1,059	30.1%
	average = 2	1,095	31.1%
	poor/very poor = 3	1,363	38.8%
Depression	with depression = 1	876	24.8%
	no depression = 0	2,663	75.2%
Anxiety	with anxiety = 1	441	12.5%
	no anxiety = 0	3,098	87.5%
Cognitive impairment (CI)	with CI = 1	1,958	55.9%
	without CI = 0	1,542	44.1%
I/ADL limitations	mild = 1	464	13.1%
	moderate = 2	1,632	40.8%
	severe = 3	1,443	46.1%
**Family endowment**			
Self-rated financial status	very rich/rich/average = 1	3,016	86.3%
	poor/very poor = 0	480	13.7%
Annual household income	yuan (logarithm; continuous numerical variable)	9.891	1.708
Cohabiting family member	number (continuous numerical variable)	2.5	1.714
Number of children who frequently visited	no children = 1	127	3.7%
	one child = 2	498	14.7%
	more than one child = 3	2,762	81.5%
Primary caregiver	spouse = 1	409	11.6%
	son and daughter-in-law = 2	2,042	57.7%
	daughter and son-in-law = 3	728	20.6%
	other people = 4	360	10.2%
Hours of care received in the past week	≤ 24 h = 1	1,393	39.4%
	25–96 h = 2	1,186	33.5%
	97–168 h = 3	960	27.1%
Cost of care in the past week	yuan (logarithm; continuous numerical variable)	4.258	2.731
**Access to health care services**			
	yes = 1	165	4.7%
	no = 0	3,330	95.3%
**Access to community-based long-term care services**			
	number (continuous numerical variable)	1.86	2.157

In terms of family endowment, the majority of disabled older adults (86.3%) reported that compared with people around them, their family's financial status was at the average level and above, and the mean of their household income (log-transformed) was 9.891. Moreover, they had an average number of cohabiting family members of 2.5, the majority of them (81.5%) had more than one child visited, most of them (57.7%) received care from their son and daughter-in-law, nearly 40% of the respondents received ≤ 24 h of care in the past week and the mean cost of care in the past week was 4.258. Furthermore, few of the respondents (4.7%) could not access health care services when necessary, and the mean number of types of CBLTCS was relatively low (1.86).

Table 2 presents the results of the percentage distribution of the respondents' perceived caregivers' willingness to provide care via their characteristics. Among the sociodemographic characteristics, place of residence, education level and financial independence significantly affected the respondents' perceived caregivers' willingness to care. For health-related data, self-rated health, anxiety, cognitive function and I/ADL limitations were significantly associated with the caregiver's willingness to provide care. In terms of family endowments, self-rated financial status, annual household income, number of children who frequently visited and hours of care received in the past week significantly influenced the respondents' perceived caregivers' willingness to care. Furthermore, access to health care services and CBLTCS were both significantly related to the respondents' perceived caregivers' willingness to provide care.

**Table 2:** Respondents' perceived caregivers' willingness by their characteristics.

**Variable**	**Willing to and provide adequate care**	**Willing to but need respite care**	**Unwilling to care or without patience**	**χ2**	***P***
**Sociodemographic attributes**					
Age				5.046	0.08
65–79	229 (85.9%)	17 (6.6%)	10 (3.9%)		
80–99	1,613 (91.6%)	113 (6.3%)	37 (2.1%)		
≥ 100	1,358 (90.4%)	118 (7.9%)	26 (1.7%)		
Gender				4.484	0.106
Male	1,065 (92.1%)	66 (5.7%)	25 (2.2%)		
Female	2,153 (90.3%)	182 (7.6%)	48 (2.0%)		
Place of residence				8.075	0.018
City/town	1,822 (92.1%)	118 (6.0%)	38 (1.9%)		
Rural	1,396 (89.4%)	130 (8.3%)	35 (2.2%)		
Education level				6.879	0.032
Illiterate	2,017 (90.3%)	173 (7.7%)	43 (1.9%)		
Primary school	967 (91.7%)	66 (6.3%)	22 (2.1%)		
Junior high and above	234 (93.2%)	9 (3.6%)	8 (3.2%)		
Marital status				4.92	0.085
Currently married	572 (89.0%)	52 (8.1%)	19 (3.0%)		
Divorced/widowed/single	2,618 (91.5%)	189 (6.6%)	54 (1.9%)		
Financial independence				8.614	0.013
Yes	644 (93.7%)	33 (4.8%)	10 (1.5%)		
No	2,495 (90.1%)	212 (7.7%)	61 (2.2%)		
**Health-related data**					
Self-rated health				61.66	< 0.001
Very good/good	1,007 (95.1%)	42 (4.0%)	10 (0.9%)		
Average	1,008 (92.1%)	70 (6.4%)	17 (1.6%)		
Poor/very poor	1,084 (86.9%)	133 (9.8%)	46 (3.4%)		
Depression				2.957	0.228
With depression	785 (89.6%)	68 (7.8%)	23 (2.6%)		
No depression	2,433 (91.4%)	180 (6.8%)	50 (1.9%)		
Anxiety				31.609	< 0.001
With anxiety	375 (85.0%)	43 (9.8%)	23 (5.2%)		
No anxiety	2,843 (91.8%)	205 (6.6%)	50 (1.6%)		
Cognitive impairment (CI)				26.547	< 0.001
With CI	1,738 (88.8%)	169 (8.6%)	51 (2.6%)		
Without CI	1,446 (93.8%)	76 (4.9%)	20 (1.3%)		
I/ADL limitations				24.441	< 0.001
Mild	422 (90.9%)	36 (7.8%)	6 (1.3%)		
Moderate	1,352 (93.7%)	62 (4.3%)	29 (2.0%)		
Severe	1,444 (88.5%)	150 (9.2%)	38 (2.3%)		
**Family endowment**					
Self-rated financial status				176.935	< 0.001
Very rich/rich/average	2,821 (93.5%)	151 (5.0%)	44 (1.5%)		
Poor/very poor	359 (74.8%)	94 (19.6%)	27 (5.6%)		
Annual household income	9.924 (1.718)	9.499 (1.737)	9.923 (1.275)	25.837	< 0.001
Cohabiting family member	2.51 (1.17)	2.44 (1.708)	2.75 (1.779)	3.103	0.212
Number of children who frequently visited				14.602	0.001
No children	104 (81.9%)	18 (14.2%)	5 (3.9%)		
One child	442 (88.8%)	44 (8.8%)	12 (2.4%)		
More than one child	2,539 (91.9%)	171 (6.2%)	52 (1.9%)		
Primary caregiver				2.07	0.355
Spouse	363 (88.8%)	38 (9.3%)	8 (2.0%)		
Son and daughter-in-law	1,865 (91.3%)	127 (6.2%)	50 (2.4%)		
Daughter and son-in-law	665 (91.3%)	53 (7.3%)	10 (2.4%)		
Other people	325 (90.3%)	30 (8.3%)	5 (1.4%)		
Hours of care received in the past week				9.91	0.007
≤ 24 h	1,260 (90.5%)	96 (6.9%)	37 (2.7%)		
25–96 h	1,068 (90.1%)	88 (7.4%)	30 (2.5%)		
97–168 h	890 (92.7%)	64 (6.7%)	6 (0.6%)		
Cost of care in the past week	4.206 (2.742)	4.503 (2.635)	4.324 (2.825)	1.655	0.437
**Access to health care services**					
				52.247	< 0.001
Yes	3,055 (91.7%)	215 (6.5%)	60 (6.8%)		
No	125 (75.8%)	28 (17%)	12 (7.3%)		
**Access to community-based long-term care services**					
				6.737	0.041
	1.89 (2.175)	1.57 (1.974)	1.52 (1.851)		

Table 3 shows the results from the multiple logistic regression analysis. Among sociodemographic characteristics, the factor associated with the perceived caregivers' willingness to provide care was the place of residence. Disabled older adults who lived in rural areas (rural vs. city/town, OR = 1.378, *P* = 0.039) were more likely to perceive that their caregivers were willing to care for them but needed respite care than willing to and provide adequate care. In health-related data, I/ADL limitations, CI and anxiety were significantly associated with the perceived caregiver's willingness to provide care. Respondents with moderate functional limitations (moderate vs. severe, OR = 0.631, *P* = 0.014) were less likely to consider that their caregivers were willing to but needed respite care than willing to and provide adequate care. Those participants with CI (yes vs. no, OR = 1.564, *P* = 0.018) were more likely to think that their caregivers needed respite care than willing to and provide adequate care. However, those participants with anxiety (yes vs. no, OR = 3.338, *P* = 0.001) were more likely to perceive that their caregiver was unwilling to care for them or lacked patience than willing to and provide adequate care. Regarding family endowment factors, respondents with no children who had frequently visited (no children vs. more than one, OR = 1.855, *P* = 0.038) were more likely to think that their caregivers needed respite care than willing to and provide adequate care. However, those participants who rated their financial status as being poor were more likely to believe that their caregivers needed respite care (poor/poorer vs. average/rich/richer, OR = 4.052, *P* < 0.001) or that their caregivers were reluctant to care (poor/poorer vs. average/rich/richer, OR = 3.681, *P* < 0.001) than willing to and provide adequate care. Those participants who received fewer hours of care in the past week (≤ 24 h vs. 97–168 h/25–96 h vs. 97–168 h; OR = 6.205, OR = 4.367; *P* < 0.001, *P* = 0.004) were more likely to believe that their caregivers were reluctant to provide care or lacked patience than willing to and provide adequate care. In terms of accessibility of public services, those participants who had poor access to health care services (no vs. yes, OR = 3.069, *P* = 0.004) were more likely to believe that their caregivers were unwilling to care for them or lacked patience than willing to and provide adequate care. However, access to CBLTCS was not significantly associated with the perceived caregiver's willingness to provide care.

**Table 3:** Multiple logistic regression analysis of factors associated with perceived caregivers' willingness to provide care among disabled older adults.

**Variable**	**Willing to but need respite care**			**Unwilling to care or without patience**		
	***B***	**Sig**.	**Exp (B)**	***B***	**Sig**.	**Exp (B)**
**Sociodemographic attributes**						
**Age** (Ref: 65–79)						
80–99	0.483	0.149	1.621	−0.328	0.515	0.720
≥ 100	0.711	0.054	2.035	−0.715	0.214	0.489
**Gender** (Ref: male)						
Female	0.318	0.098	1.374	0.337	0.303	1.401
**Place of residence** (Ref: city /town)						
Rural	0.321	0.039	1.378	0.090	0.749	1.095
**Education level** (Ref: junior high and above)						
Illiterate	0.598	0.167	1.818	−0.509	0.373	0.601
Primary school	0.483	0.259	1.621	−0.386	0.490	0.680
**Marital status** (Ref: currently married)						
Divorced/widowed/single	−0.375	0.219	0.687	−0.721	0.106	0.486
**Financial independence** (Ref: yes)						
No	−0.227	0.341	0.797	0.253	0.578	1.288
**Health-related data**						
**Self-rated health** (Ref: very good/good)						
Poor/very poor	0.318	0.142	1.374	0.722	0.078	2.058
Average	0.271	0.223	1.311	0.251	0.574	1.285
**Depression** (Ref: no depression)						
With depression	−0.145	0.456	0.865	−0.390	0.274	0.677
**Anxiety** (Ref: no anxiety)						
With anxiety	0.327	0.146	1.387	1.205	0.001	3.338
**Cognitive impairment** (Ref: without CI)						
With CI	0.447	0.018	1.564	0.624	0.070	1.866
**I/ADL limitations** (Ref: severe)						
Mild	0.239	0.337	1.270	−0.794	0.180	0.452
Moderate	−0.460	0.014	0.631	0.133	0.666	1.143
**Family endowment**						
**Self-rated financial status** (Ref: very rich /rich/average)						
Poor/very poor	1.399	< 0.001	4.052	1.303	< 0.001	3.681
**Annual household income** (continuous numerical variable)	−0.039	0.339	0.962	0.162	0.126	1.176
**Cohabiting family member** (continuous numerical variable)	0.031	0.482	1.031	0.063	0.396	1.065
**Number of children who frequently visited** (Ref: more than one child)						
No children	0.618	0.038	1.855	0.711	0.180	2.036
One child	0.080	0.685	1.083	0.171	0.635	1.187
**Primary caregiver** (Ref: other people)						
Spouse	0.621	0.116	1.861	0.087	0.923	1.091
Son and daughter-in-law	−0.016	0.951	0.984	1.343	0.070	3.829
Daughter and son-in-law	0.257	0.377	1.293	0.959	0.226	2.610
**Hours of care received in the past week** (Ref: 97–168 h)						
≤ 24 h	0.259	0.208	1.296	1.825	< 0.001	6.205
25–96 h	0.164	0.400	1.178	1.474	0.004	4.367
**Cost of care in the past week** (continuous numerical variable)	0.026	0.365	1.026	0.010	0.845	1.010
**Access to health care services (Ref: yes)**						
No	0.444	0.088	1.559	1.121	0.004	3.069
**Access to community-based long-term care services** (continuous numerical variable)						
	−0.047	0.222	0.954	−0.108	0.176	0.898
Constant	−4.382	< 0.001		−8.369	< 0.001	
Nagelkerke *R*^2^	0.167					

“Willing to and provide adequate care” was used as the reference group.

## 4. Discussion

This study examined the factors associated with perceived caregivers' willingness to provide care among older adults with disabilities in China based on the 2018 wave of CLHLS data. In general, we found that the majority of disabled older adults had a positive attitude toward their caregivers' willingness to care and the care that they received; however, 7.0% of them were concerned about their caregivers' ability to handle care and need respite care. Studies have found that perceived need for respite care indicated that their caregivers could no longer cope (33) and that they would need assistance and support (such as domestic help and short breaks) rather than taking over the care (34). It is crucial to identify those individuals at high risk and offer them respite care options; otherwise, it is likely that these informal caregivers' initial willingness to provide care will decline. Moreover, a small number of disabled older people felt that their caregivers were reluctant to care or lacked patience. Although there may be discordance between the actual and perceived carers' willingness to care, we should still pay attention to this result. Existing research has confirmed that reluctance to care (as the opposing idea to willingness) has a negative impact on the caregivers' mental health (e.g., feeling resentful and exhibiting depression, etc.), quality of care that is received (e.g., premature institutionalization and neglect/abuse, etc.) and family conflict (21, 35–37).

In the bivariate analysis, most of the included factors in the study were significantly associated with the respondents' perceived caregivers' willingness. To some extent, this analysis identified useful information; however, it only considered the influence of a single factor. Therefore, we used a multinomial logit model to identify factors related to perceived caregivers' willingness to care among disabled older adults and to draw our conclusions accordingly.

Compared to their urban counterparts, this study found that older adults living in rural areas were more likely to perceive that their caregivers were helpful but needed respite care than willing to and provide adequate care. Although average household income has risen considerably in China over the last decades, urban–rural gaps still exist. In addition, due to the urban rural dual social security system in China, older rural residents received lower pension support (38). Moreover, there are urban–rural disparities in CBLTCS (39). Furthermore, with the accelerated process of urbanization, massive younger populations have traveled to cities to earn income (40). Given the poor ability to pay for pension services, insufficient supply of CBLTCS and the shrinkage of potential care providers in rural areas, it is not easy for informal caregivers to care for disabled rural older adults with these disadvantages; thus, they are more likely to need additional help.

It has been well documented that patients' level of I/ADL and the number of neuropsychiatric symptoms were significantly associated with caregivers' distress and burden, and it was assumed that these types of symptoms were particularly demanding for care (41–44). In that sense, it is natural to understand that respondents with severe disabilities or CI were more likely to report that their caregivers needed respite care.

Previous studies have found that family caregivers generally expect little formal help, and they may look forward to some help from family members, relatives or friends (20, 37). Given the deep-rooted Confucian cultural influences in China, adult children are the most preferred support providers. For those individuals with no children who visited them often, older adults usually have to rely on their spouses; however, spouse caregivers are usually older adults themselves, they were more likely to report moderate or severe caregiver burden (22). When the more “natural” caregiver, like the spouse or child were not available or had little inclination to contribute, disabled older adults have to lean on supporters outside of their family, namely non-kin caregivers. The reasons for providing care are varied, such as to reciprocate friendship/kindness, feel compassion to the care recipients, etc. However, non-kin care recipients often report of feeling guilty for their dependence on I/ADL tasks or of worrying about being a burden on their friends or neighbors (45). In that sense, it is natural to understand that respondents with no children who had frequently visited were more likely to perceive that their caregivers needed respite care.

In addition, the results showed that respondents who rated their financial status as being poor faced a high risk that their caregivers were unable to/unwilling to take care of them. This result corroborates the findings of previous literature underlying the negative effect that low income may have on caregiving willingness (20, 21). Studies have shown that poverty affects caregiving in many ways, and low-income families who are unable to purchase commercial services as supplements or alternatives may resort to caregiving with no options rather than actively choosing the role (20). Additionally, they may live in neighborhoods or rural areas that lack available formal services; moreover, they may encounter barriers that impede their access to formal care (46). Furthermore, those individuals who were not poor may have intended to use economic stimulus or property transfer (e.g., inheritance) in exchange for contact and care from their children (47, 48). After all, 78.3% of the respondents were cared for by their children and their children's spouses.

The findings suggest the urgent need for more support to be provided for caregivers of those individuals with socioeconomic disadvantages (living in rural areas, being poor and with no children who frequently visited) or with high demand (with severe disabilities or CI). Apart from expanding the scale and coverage of LTCI support, designing a more tailored, user-friendly and innovative LTCI service scheme is essential for addressing the needs of this population, so as to increase the options available to these caregivers in obtaining supportive physical and emotional assistance. In addition, private sector and non-profit institutions are encouraged to participate in providing diversified and selective services for older adults and their families.

Anxiety has been linked to an increased demand for personal assistance and unmet needs for I/ADL (41); however, little research exists addressing the relationship between anxiety and caregiving willingness. Our research identified that respondents with anxiety symptoms were more likely to perceive that their caregivers were unwilling to care for them. This may be due to the fact that when the older adults perceived the reluctance of the caregivers, it would make them feel powerless and anxious. Caregiving is primarily a relational experience (49), and willingness to care develops out of close relationships, whereas reluctance to care overshadows the carer-care recipient relationship (21, 50). There is abundant literature that has consistently documented that high-quality relationships between care recipients and caregivers serve a protective role for care recipients, whereas the loss of closeness or trust can lead to the negative emotions of older adults (51, 52). Due to the fact that this was a cross-sectional study, we could not determine a causal inference.

It is noteworthy that, compared to those individuals who have been cared for the highest amount of time, disabled older adults who received fewer hours of care were more likely to feel that their caregivers were reluctant or lacked patience. However, the results demonstrated that self-rated health, the degree of disability and CI did not have statistical significance with the reluctance to care. Based on this situation, we may assume that because of this reluctance, caregivers will devote less time to attending to the needs of disabled older adults. The findings of this study are in conjunction with other studies that have identified that reduced willingness to care was associated with lower investment in caregiving (36).

This study indicated that those individuals who could not access health care services when needed were at a high risk of having caregivers with reluctance or a lack of patience. Factors related to the accessibility of health care services were comprehensive and complex, and previous research has found that the relevant factors included the uneven distribution of resources, inconvenience in mobility, economic burden and lack of help from informal caregivers (53–56). Mai (57) argued that policies such as full health care insurance coverage, telemedicine and the family doctor system (to a great extent) could realize geographic and service accessibility. However, help from informal caregivers always plays an important role in this scenario. Therefore, it could be speculated that when disabled older adults are ill, they cannot obtain adequate health care services through the help of their family caregivers, nor can they obtain attentive daily care from their family members with a degree of willingness.

The study found that caregivers' reluctance to care was not rare and factors related to it were very complex and complicated. So we cannot pretend that all caregivers are willing to take care of disabled older adults, and try not to stand on the moral high ground to view this issue, what we can do is seek to convert caregiving reluctance into a more measurable concept, uncover the underlying causes and mechanisms, and identify their preferences and needs, and then provide targeted and preference-oriented support for disabled older people and their caregivers.

However, there were some limitations in our study. First, informal carers' willingness to care was only measured from the perspective of the care recipients due to data restrictions. Study results may not objectively reflect the actual caregivers' willingness to care. Additionally, concordance between the actual and perceived carers' willingness to care needs to be determined in future research. However, it can be difficult to measure the caregivers' willingness in a straightforward manner due to reporting bias; moreover, considering that informal caregivers are not routinely assessed in China, such variables from the perspective of the care recipients could indirectly reflect the problem and guide the formation of related policies. Second, there's no measures related to the caregiver's situation in the study due to data restrictions as well. However, caregiver-specific situation, e.g., employment status, may impact the associations between circumstances and care-recipient perceptions of caregivers' willingness to care. Third, it should be noted that the cross-sectional nature of this study may not be applicable for making causal inferences between the variables, as was mentioned earlier. However, causality between variables (e.g., care recipients' anxiety and carers' willingness to care) and dynamic changes of carers' willingness to care over different stages of the caring career needs to be examined in future research by using a different methodological approach (e.g., longitudinal protocols).

## 5. Conclusion

This study found that living in rural areas, being poor, with no children who frequently visited, severe disabilities or CI were positively associated the care recipients' perception that caregivers needed respite care. While anxiety symptoms, a lower amount of care time, poor self-rated financial status and poor accessibility to health care services were significantly associated with care recipients' perception of caregivers' reluctance to care. This study highlights the awareness of monitoring informal carers' willingness to care or capability to enact caring tasks. Furthermore, the results can inform policies aimed at developing and implementing tailored, user-friendly and innovative support programs for vulnerable disabled older adults and their family caregivers, so as to increase their wellbeing and enlarge the caring capacity of older adults' caregiving networks.

## Data availability statement

Publicly available datasets were analyzed in this study. This data can be found at: CLHLS_2018_cross_sectional_dataset_15874.rar; https://opendata.pku.edu.cn/file.xhtml?fileId=10356&amp;version=2.1.

## Author contributions

CS wrote the manuscript and conducted the data analysis. DM contributed in writing the manuscript and participated in the final review of the paper. All authors contributed to the article and approved the submitted version.
